# Machine learning derived development and validation of extracellular matrix related signature for predicting prognosis in adolescents and young adults glioma

**DOI:** 10.1038/s41598-025-13547-6

**Published:** 2025-08-07

**Authors:** Pancheng Wu, Yi Zheng, Wei Wu, Beichen Zhang, Yichang Wang, Mingjing Zhou, Ziyi Liu, Zhao Wang, Maode Wang, Jia Wang

**Affiliations:** 1 Department of Neurosurgery, The First Affiliated Hospital of Xi’an Jiaotong University, Xi’an, China; 2 Center of Brain Science, The First Affiliated Hospital of Xi’an Jiaotong University, Xi’an, China; 3 Department of Clinical Oncology, Xijing Hospital, The Fourth Military Medical University, Xi’an, China; 4 Department of Clinical Medicine, Xi’an Jiaotong University Health Science Center, Xi’an, China; 5 Department of Bone and Joint Surgery, The Second Affiliated Hospital of Xi’an Jiaotong University, Xi’an, China

**Keywords:** Adolescents and young adults, Glioma, Machine learning, Prognosis, Immunotherapy, CNS cancer, Tumour biomarkers, Cancer models, Data mining, Machine learning, Predictive medicine

## Abstract

The mortality rates have been increasing for glioma in adolescents and young adults (AYAs, aged 15–39 years). However, current biomarkers for clinical assessment in AYAs glioma are limited, prompting the urgent need for identifying ideal prognostic signature. Extracellular matrix is involved in the development of tumors, while their prognostic significance in AYAs glioma remains unclear. By an integrated machine learning workflow and circuit training and validation procedure, we developed a machine learning-derived prognostic signature (MLDPS) based on 1,026 extracellular matrix-related genes and 3 AYAs glioma cohorts. MLDPS exhibited robust and consistent predictive performance in overall survival and could serve as an independent prognostic factor for AYAs glioma. Simultaneously, MLDPS outperformed previous 89 published prognostic signatures and traditional clinical characteristics, confirming the robust predictive capability. Besides, MLDPS had the potential to stratify prognosis in patients with other cancer types. In addition, the tumor microenvironment between high and low MLDPS groups displayed different patterns while more tumor-infiltrating immune cells were observed in high MLDPS group. Additionally, patients in low MLDPS group had significantly prolonged survival when received immunotherapy in cancers including glioblastoma, urothelial carcinoma and melanoma. Overall, our study proposes a promising signature, which can be utilized for clinicians to evaluate prognosis and might provide individualized clinical management for AYAs glioma.

**Supplementary Information:**

The online version contains supplementary material available at 10.1038/s41598-025-13547-6.

## Introduction

As one of the most prevalent tumors in central nervous system^1^(CNS), gliomas can occur across different age groups including adolescents and young adults (AYAs, aged 15–39). In 2020, the Cancer Statistics estimated that there would be nearly 89,500 new AYAs cancer diagnoses and 9,270 cancer deaths in the United States, among which brain tumor is the leading cause of cancer death in AYAs men and the second leading cause in AYAs women^2^. The fifth edition of World Health Organization tumor classification for CNS (WHO CNS5) has introduced several molecular markers for accurate diagnosis in CNS tumors and made detailed distinction between the pediatric- and adult-type gliomas, which highlighted the biological and clinical differences between different age groups^3^. However, the prognostic implications with molecular features in AYAs glioma remain poorly elaborated^4^. The mortality rates have been increasing for AYAs glioma in recent years^4,5^. Currently, AYAs glioma treated in adult centers are usually treated according to the adult guidelines, while those treated in pediatric centers are often treated with pediatric scheme due to the lack of literature focusing on the precise stratification and treatment for AYAs glioma^6^. Therefore, it is imperative to develop prognostic signature to survival stratification, prognosis prediction and precisely individualized management for AYAs glioma.

The extracellular matrix (ECM), served as an essential regulator of cell behavior, is mainly composed of collagen, proteoglycans, laminin and fibronectin^7–9^. As a highly dynamic structure, ECM plays important role during organism development, tissue homeostasis, cell fate determination, cell migration and proliferation and modulation of the microenvironment^9–11^. Whereas the dysregulation in ECM could lead to development of cancer and has been recognized as remarkable feature of cancer. The ECM and its remodeling by the cells in the tumor microenvironment (TME) including stromal, immune and cancer cells have been involved in facilitating the progression in cancers^12–14^. For instance, through ECM remodeling, cancer-associated fibroblasts-derived HAPLN1 could promote the invasion in gastric cancer via increasing the fiber alignment and decreasing the width, length, number and density of the fibers^15^. The abnormal ECM has been linked to the relapse and resistance to chemotherapy in breast cancer, thus affecting the prognosis^16^. In the TME of glioblastoma, mesenchymal stem-like cells educated by glioblastoma were capable of promoting glioblastoma infiltration by ECM remodeling^17^. Recently, increasing studies have focused on the prognostic roles of ECM-related genes in cancers and developed risk stratification model in order to make precision strategies for clinical management toward patients^18–20^. However, it remains unclear how the ECM-related genes influencing the prognosis of AYAs gliomas.

In this study, we developed a machine learning-derived prognostic signature (MLDPS) via circuit training and validation procedures in different cohorts through 65 combinations of machine learning algorithms for AYAs glioma. In different cohorts, MLDPS exhibited robust and consistent predictive performance in overall survival (OS). Additionally, MLDPS also possessed a remarkably superior performance compared with 89 published prognostic signatures as well as common clinical features. Patients in different MLDPS groups had distinct fraction of tumor-infiltrating immune cells. Furthermore, MLDPS had the potential to forecast the outcome in multiple cancer types including glioma in other age groups and predict the prognosis for patients received immunotherapy. Overall, MLDPS could serve as a signature with prognostic potential in evaluating prognosis and individualized clinical management for AYAs glioma.

## Methods

### Data acquisition and preprocessing

We collected datasets from the friendly web tool named UCSC Xena portal (https://xenabrowser.net/datapages/)^21^ and Chinese Glioma Genome Atlas^22^ (CGGA, https://www.cgga.org.cn/) via the following criteria: (1) the transcriptomic data type should be RNA sequencing; (2) with complete survival information; (3) more than 50 patients. Then, three cohorts including TCGA-GBMLGG cohort, CGGA-693-cohort and CGGA-325 cohort were included. Finally, after selecting patients aged in 15–39 and overall survival (OS) more than 30 days, a total of 578 patients from three glioma cohorts including The Cancer Genome Atlas-lower grade glioma and glioblastoma (TCGA-GBMLGG, *n* = 223), CGGA-693^23^ (*n* = 238) and CGGA-325^24^ (*n* = 117) were enrolled for subsequent analysis. In addition, we obtained a total of 1,026 extracellular matrix-related genes from previous literature^25^ and summarized these genes in supplementary Table 1. The transcriptome data of the normal brain from the Genotype-Tissue Expression (GTEx) were also acquired from UCSC Xena. These datasets have been summarized in supplementary Table 2 and the flowchart of this section was displayed in supplementary Fig. 1.

### Development of the MLDPS

The establishment pipelines of MLDPS were composed of four steps. (1) First, we obtained the differentially expressed genes (DEGs) between AYAs glioma in TCGA-GBMLGG (*n* = 223) and control samples in GTEx-brain (*n* = 1148) like previous studies^26,27^ via DESeq2 package^28^ with |log2FC| > 1 and adjusted P value < 0.05. The intersection genes between 1,026 ECM-related genes and DEGs were considered as ECM-related DEGs. Then, we further took the intersection genes among the three cohorts and ECM-related DEGs. (2) Next, based on the above intersection genes, univariate Cox regression analysis was employed to identify prognostic genes in TCGA, CGGA-693 and CGGA-325 cohort, respectively. Based on this step, we used each cohort as training cohort and the others as validation cohorts to avoid overfitting caused by focusing on just one specific training cohort like TCGA in previous studies^29–31^. Specifically, for TCGA cohort training procedure, we extracted the expression of prognostic genes identified in TCGA among all three cohorts and merged their corresponding survival data for preparing the input data for subsequent analysis. The CGGA-693 and CGGA-325 training procedures were the similar with the TCGA training procedure. (3) Then, an integrated machine learning workflow^32–34^ was applied to develop MLDPS. This workflow was consisted of ten prevalent machine learning algorithms including random survival forest (RSF), Stepwise Cox, elastic network (Enet), CoxBoost, partial least squares regression for Cox (plsRcox), least absolute shrinkage and selection operator (Lasso), Ridge, gradient boosting machine (GBM), supervised principal components (SuperPC) and survival support vector machine (Survival-SVM). These algorithms were combined into 65 machine learning algorithm combinations to fit a prognostic model. (4) Finally, we calculated the C-index for each model across all cohorts when setting TCGA, CGGA-693 and CGGA-325 as training cohort, respectively. The optimal model was determined by the highest average C-index.

### Evaluation of the prognostic value and clinical significance of MLDPS

Patients in CGGA-693, CGGA-325 and TCGA cohort were categorized into high and low MLDPS groups according to median value. Kaplan-Meier survival analysis was conducted to evaluate the prognostic value of MLDPS. Moreover, we performed receiver-operator characteristic (ROC) analysis through timeROC package and calculated the area under the curve (AUC) value to assess the predictive performance of MLDPS. Afterward, we also merged MLDPS with clinical and molecular characteristics like age, gender, grade and IDH mutation status to determine whether MLDPS could serve as an independent prognostic factor via univariate and multivariate Cox regression analyses in the three AYAs glioma cohorts, respectively. Besides, we compared the differences in MLDPS among different clinical characteristics subgroups such as age, gender, grade, IDH and 1p/19q status. Moreover, we performed survival analysis in subgroups using Kaplan-Meier analysis. Additionally, we also explored the application value of MLDPS across pan-cancer level. The RNA-seq and survival data of TCGA pan-cancer were downloaded from UCSC Xena portal.

### Collection and evaluation of published prognostic signatures in glioma

With the increasing attention focusing on the precise treatment and stratified management for glioma, a considerable number of prognostic signatures in glioma have been constructed in recent years. To compare the predictive capability of MLDPS with these published models, we comprehensively searched the published mRNA prognostic signatures in PubMed up to May 31, 2024. Published signatures focused on glioma rather than just low-grade glioma or glioblastoma and included the exact prediction formula were selected for further study. Subsequently, we calculated the risk score for every patient in the three cohorts following the formula and systematically evaluated the performance of the enrolled studies in AYAs glioma through C-index.

### Pathway enrichment analysis

Gene set enrichment analysis (GSEA) was applied to elucidate the potential biological processes and pathways in high and low MLDPS groups. Firstly, we used the DESeq2 package to perform differential analysis and ranked the genes based on the log_2_FoldChange value^28^. Next, the clusterProfiler and enrichplot package were employed to identify and visualize the biological processes (BP) and the pathways with the reference gene sets c2.cp.v2024.1.Hs.symbols and c5.go.bp.v2024.1.Hs.symbols obtained from MSigDB database^35–38^. Additionally, we utilized the clusterProfiler package to investigated the potential biological processes and pathways enriched in MLDPS^39^.

### Correlation analysis between MLDPS and tumor microenvironment

The ESTIMATE algorithm was utilized to calculate the stromal and immune scores which represent the infiltration of stromal and immune cells within the tumor and evaluate tumor purity for each patient^40^. For evaluation of tumor immune environment landscape, we used the ssGSEA algorithm and CIBERSORTx tool. Specifically, the GSVA package was utilized to assess the levels of 28 infiltrating immune cells using ssGSEA method based on the immune metagenes from previous study^41,42^. Moreover, the CIBERSORTx web-tool (https://cibersortx.stanford.edu/) was used to evaluate 22 immune cells in different MLDPS groups^43^.

### Prediction of immunotherapy response

Immunotherapy cohorts with complete survival information were utilized to predict immunotherapy response. We obtained the transcriptome data, survival information and immunotherapy effects of the IMvigor 210 cohort^44^ through IMvigor210CoreBiologies package. The expression data, clinical information and immunotherapy results of PRJNA482620^45^, GSE91061^46^ and GSE78220^47^ cohorts were downloaded from the Tumor Immunotherapy Gene Expression Resource portal^48^ (http://tiger.canceromics.org/). We calculated the MLDPS score for every patient in these cohorts to explore the impact of MLDPS on immunotherapy. The survival differences in patients received anti PD-1/PD-L1 therapy between high and low MLDPS groups were assessed using Kaplan-Meier method.

### Statistical analysis

All data preprocessing, analysis and visualization for this study were performed in R language (version 4.3.3, https://www.r-project.org/) and an integrated development environment named Rstudio (version 2024.04.0-735). Comparisons of differences between two or multiple groups were assessed using Wilcoxon test or Kruskal–Wallis test, respectively. Kaplan-Meier survival analysis with log-rank test was utilized to determine the survival differences in different groups. Heatmaps were drawn using ComplexHeatmap package^49,50^. P-value < 0.05 deemed statistically significant.

## Results

### Integrated construction of MLDPS

**Fig. 1:**
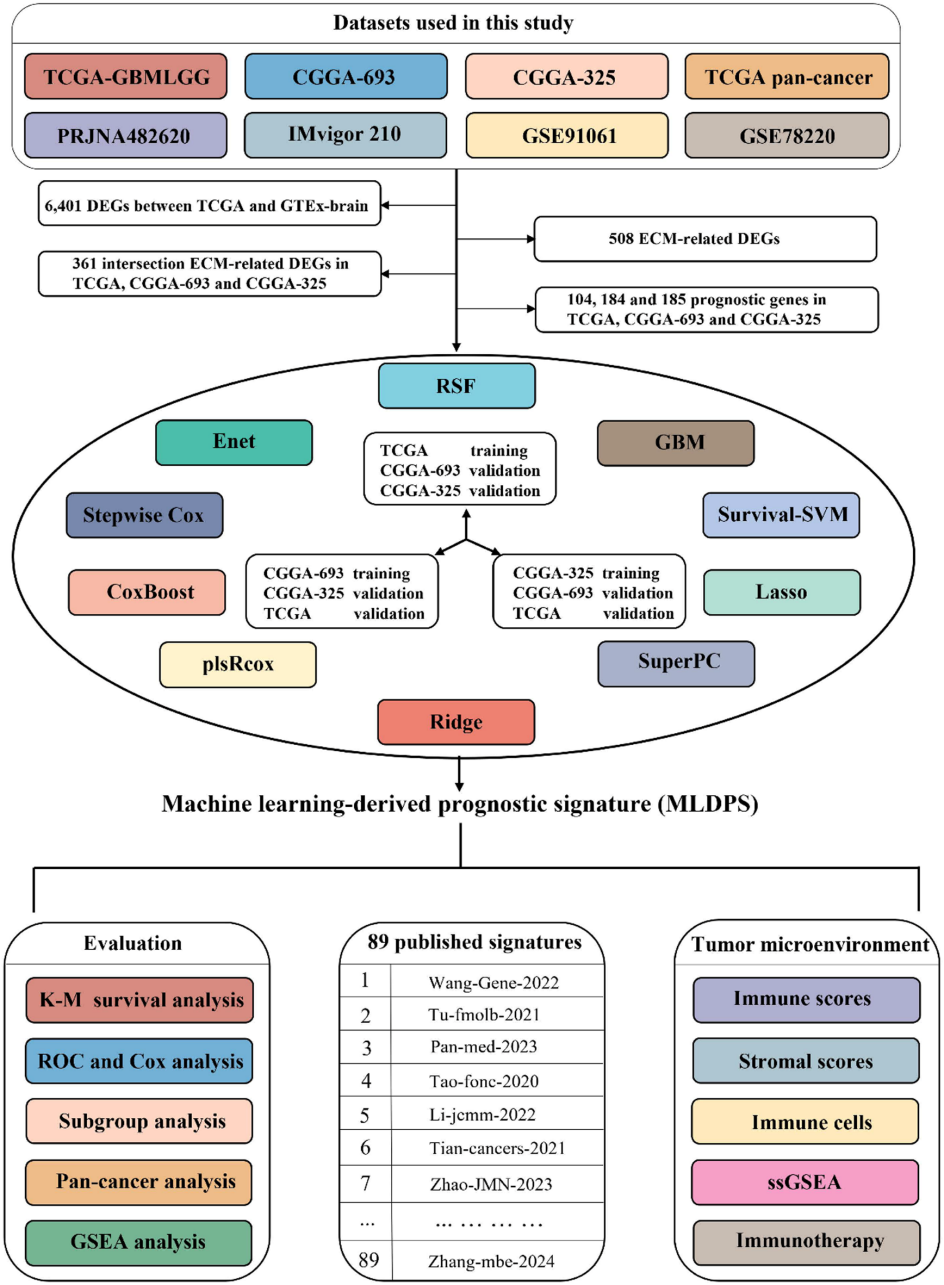
The flowchart of this study.

**Fig. 2:**
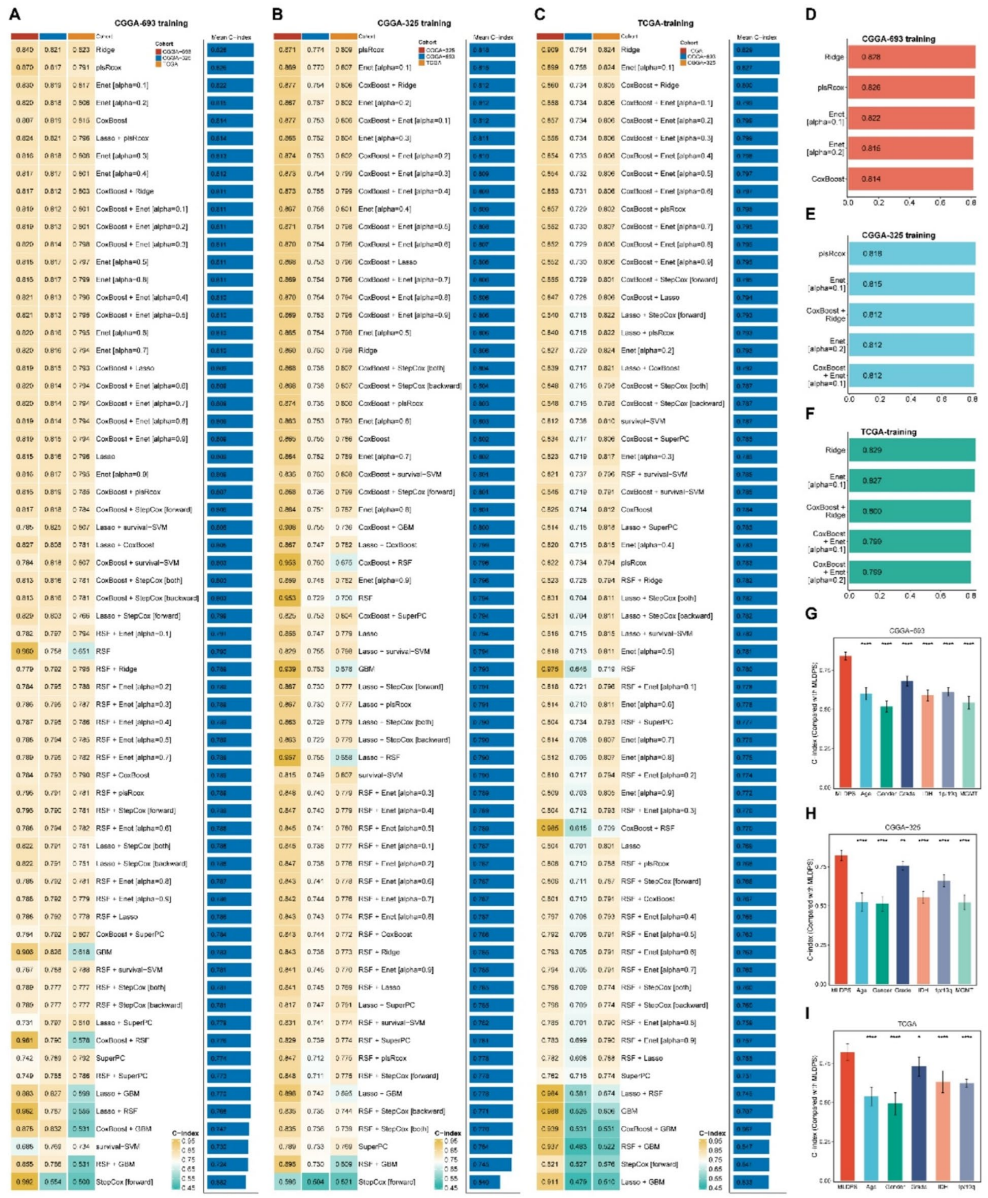
Construction of the machine learning-derived prognostic signature (MLDPS). (A) The C-index of 65 machine learning algorithms combinations in CGGA-693 training procedure. (B) The C-index of 65 machine learning algorithms combinations in CGGA-325 training procedure. (C) The C-index of 65 machine learning algorithms combinations in TCGA training procedure. (D-F) Top five average C-index in CGGA-693, CGGA-325 and TCGA training procedure, respectively. (G-I) The performance of MLDPS was compared with common clinical and molecular characteristics in CGGA-693 (G), CGGA-325 (H) and TCGA (I). *p < 0.05, **p < 0.01, ***p < 0.001, ****p < 0.0001.

The workflow of our study was illustrated in Fig. 1. With |log_2_FC| > 1 and adjusted P value < 0.05, 6,401 differentially expressed genes (DEGs) were screened out between AYAs glioma and control. After intersecting with 1,026 ECM-related genes, 508 ECM-related DEGs were identified. Next, we obtained 361 overlapped genes existed in ECM-related DEGs, TCGA, CGGA-693 and CGGA-325 cohorts. Different from previous studies, we hypothesized that each cohort had the potential to generate the optimal prognostic model when treated as the training cohort. Therefore, our study proposed an innovative circuit training and validation procedure to the machine learning workflow, which means when one cohort utilized to training the model, others were used for validation. We identified 104, 184 and 185 prognostic genes in TCGA, CGGA-693 and CGGA-325 cohorts, respectively, by univariate Cox analysis. Subsequently, we applied 65 machine learning algorithm combinations to develop the prognostic models with ten-fold cross-validation and calculated C-index for each algorithm in all cohorts. The highest average C-index in CGGA-693 training, CGGA-325 and TCGA training cohorts were 0.828 (0.823 in TCGA, 0.840 in CGGA-693 and 0.821 in CGGA-325, Fig. 2A), 0.818 (0.809 in TCGA, 0.774 in CGGA-693 and 0.871 in CGGA-325, Fig. 2B) and 0.829 (0.909 in TCGA, 0.754 in CGGA-693 and 0.824 in CGGA-325, Fig. 2C), respectively. Top five average C-index in each training cohort were shown in Fig. 2D-F. Obviously, overfitting was detected in the highest mean C-index in TCGA training session, with C-index of 0.909 in TCGA, while a C-index less than 0.8 (0.754) in external validation CGGA-693 cohort. Consequently, we selected the second highest average C-index derived from Ridge algorithm in CGGA-693 training session as the optimal model on account of all C-index were more than 0.80 in this model and defined it as MLDPS. The prognostic genes included in MLDPS and the formula for calculating MLDPS score were summarized in supplementary Table 3 and Table 4, respectively.

### Robust and consistent predictive performance of MLDPS

In clinical practice and management, several clinical and molecular characteristics such as grade, IDH status, age, gender and 1p/19q status have been applied for designing treatment regimens and prognosis prediction in glioma. Therefore, we contrasted the predictive performance of MLDPS with these features in the training and validation cohorts. As shown in Fig. 2G-I, the C-index of MLDPS was obviously higher than other features, suggesting MLDPS had obvious improved accuracy in prognosis prediction.

**Fig. 3:**
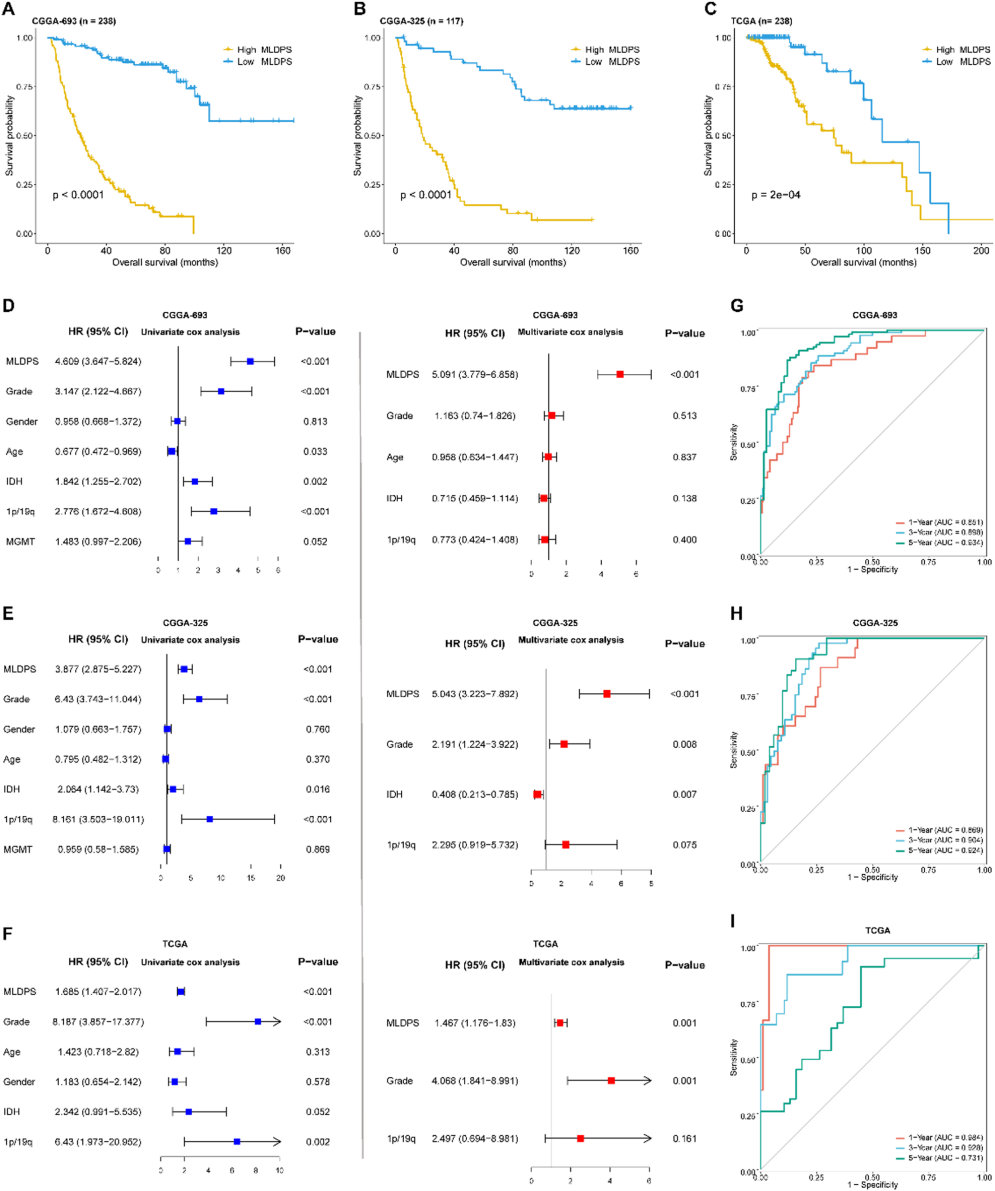
Survival analysis and predictive performance evaluation of machine learning-derived prognostic signature (MLDPS). (A-C) Kaplan-Meier survival analysis for overall survival between high and low MLDPS groups in CGGA-693 (A), CGGA-325 (B) and TCGA cohorts (C). (D-F) Univariate and multivariate Cox regression analyses regarding of MLDPS in CGGA-693 (D), CGGA-325 (E) and TCGA cohorts (F). (G-I) Time-dependent receiver-operator characteristic (ROC) analysis for predicting OS at 1-, 3- and 5-year in CGGA-693 (G), CGGA-325 (H) and TCGA cohorts (I).

In addition, patients were dichotomized into high and low MLDPS groups according to the median MLDPS score (supplementary Table 5). The Kaplan-Meier curves showed that patients in high MLDPS group had obviously dismal OS compared with low MLDPS group in CGGA-693 cohort (*p* < 0.0001, Fig. 3A). Similar results were observed in CGGA-325 (*p* < 0.0001, Fig. 3B) and TCGA cohorts (*p* = 2e-04, Fig. 3C). Additionally, univariate Cox analysis revealed that MLDPS was a risky prognostic factor in CGGA-693 cohort (HR: 4.609 [3.647–5.824], *p* < 0.001, Fig. 3D). After adjusting for common clinical characteristics like grade, IDH, 1p/19q status and age (*p* < 0.05), MLDPS still remained a remarkably risky factor for prognosis in CGGA-693 cohort (HR: 5.091 [3.779–6.868], *p* < 0.001, Fig. 3D). Consistently, the analyses in CGGA-325 and TCGA cohorts demonstrated that MLDPS could serve as an independent prognostic factor for AYAs glioma (Fig. 3E-F). Furthermore, we also evaluated the predictive performance of MLDPS using ROC analysis. The areas under ROC curve (AUC) for 1-, 3- and 5-year survival were 0.851, 0.898 and 0.934, respectively, in CGGA-693, indicating that MLDPS owns robust performance in training cohort (Fig. 3G). Additionally, similar results were witnessed in two validation cohorts, including 0.869, 0.904 and 0.924 in CGGA-325 cohort (Fig. 3H) and 0.984, 0.928 and 0.731 in TCGA cohort (Fig. 3I). Taken together, these results indicated MLDPS possessed a robust and stable performance in predicting prognosis across different independent AYAs glioma cohorts.

### The clinical significance of MLDPS

**Fig. 4:**
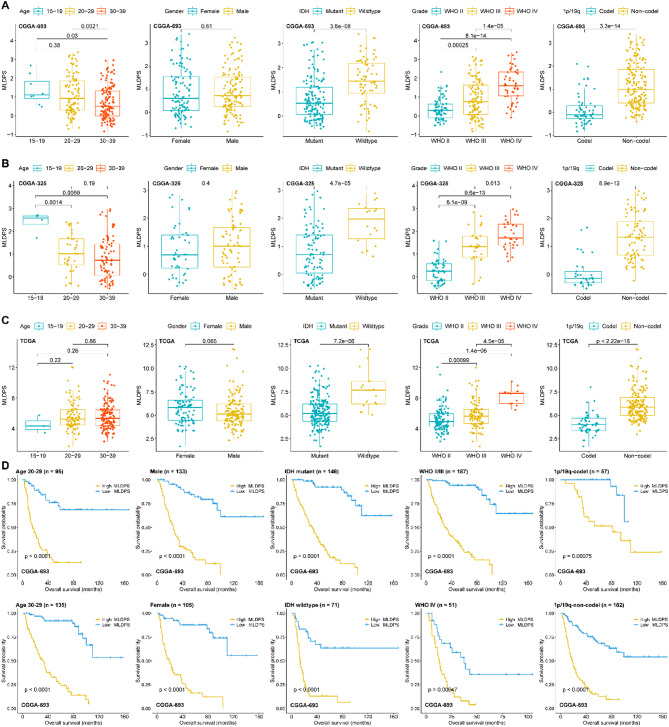
The correlation between machine learning-derived prognostic signature (MLDPS) and clinical characteristics. (A-C) The correlation between age, gender, grade, IDH status, 1p/19q status and MLDPS in CGGA-693 (A), CGGA-325 (B) and TCGA cohort (C), respectively. (D) Kaplan-Meier survival analysis for overall survival between high and low MLDPS groups in different age, gender, grade, IDH status and 1p/19q status subgroups in CGGA-693 cohort.

The results of subgroup analyses indicated that patients aged in 30–39 had lower MLDPS compared with younger patients in CGGA-693 (Fig. 4A) and CGGA-325 cohorts (Fig. 4B), while no differences were found in TCGA cohort (Fig. 4C). Besides, patients with IDH-wildtype, higher grade and 1p/19q non-codeletion had higher MLDPS in all cohorts (Fig. 4A-C). However, there were no differences in gender between high and low MLDPS groups (Fig. 4A-C). In addition, we also performed stratification survival analysis in different subgroups using Kaplan-Meier method except for the subgroups with only few patients like age 15–19 subgroups. As shown in Fig. 4D, in different groups, such as age 20–29, 30–39, male, female, IDH mutant, IDH-wildtype, WHO II/III, WHO IV, 1p/19q codeletion and non-codeletion subgroups, patients with high MLDPS had significantly worse OS than low groups in CGGA-693 cohort (all *p* < 0.05). The Kaplan-Meier curves in CGGA-325 and TCGA cohorts were similar with the above results (supplementary Fig. 2). These findings indicated that high MLDPS was associated with worse clinical behavior in AYAs glioma.

### MLDPS outperforms previous 89 published prognostic signatures

The rapid development in high-throughput sequencing has greatly facilitated the precise treatment and stratified management for patients with cancer. Numerous prognostic signatures in glioma have been developed through different algorithms such as univariate Cox analysis and Lasso algorithm based on RNA-seq or microarray data among different cohorts. However, to the best of our knowledge, we found no studies focused on prognostic signatures in AYAs glioma. The available published prognostic signatures in glioma always focused on patients across all age groups. Considering that the authors concluded that their models could predict the prognosis of patients with glioma, which included the AYAs group in our study, we decided to collect these published models focused on both lower grade gliomas and glioblastoma multiforme. Finally, we comprehensive collected 89 published mRNA prognostic signatures to compare their predictive performance in AYAs glioma with MLDPS. The detailed information of these signatures was summarized in supplementary Table 6.

**Fig. 5:**
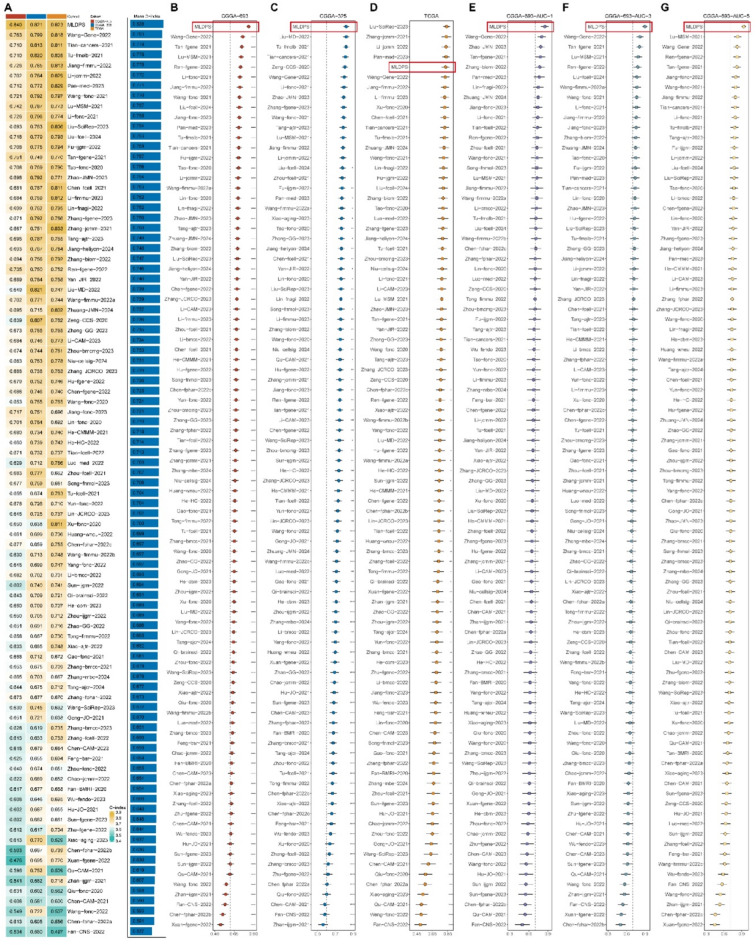
Comparisons between machine learning-derived prognostic signature (MLDPS) and 89 published prognostic signatures. (A) The C-index of 89 published signatures and MLDPS in CGGA-693, CGGA-325 and TCGA cohort. (B-D) Comparisons between C-index of MLDPS and 89 published signatures in CGGA-693 (B), CGGA-325 (C) and TCGA cohorts (D). (E-G) Comparisons between the area under the curve (AUC) values of MLDPS and 89 published signatures in predicting overall survival at 1-year (E) 3-year (F) and 5-year (G) in CGGA-693 cohort, respectively. *p < 0.05, **p < 0.01, ***p < 0.001, ****p < 0.0001.

We calculated C-index for each published signature across all cohorts and made comparison with MLDPS via compare C package. Our MLDPS exhibited the highest mean C-index 0.828 than other signatures across the three cohorts (Fig. 5A). Additionally, the C-index of MLDPS ranked first in both CGGA-693 (Fig. 5B) and CGGA-325 cohorts (Fig. 5C). In TCGA cohort, MLDPS ranked the fifth among all signatures, while the top four displayed poor performance in CGGA-693 and CGGA-325 cohorts (Fig. 5D). For example, the signatures presented by Liu et al.^51^ and Zhang et al.^52^ ranked the first and second in TCGA cohort, whereas their C-index in CGGA-693 were both less than 0.7. This overfitting in models might weaken the generalization power for clinical practice^30,34^. Furthermore, we also compared the AUC values of 1-, 3- and 5-year between MLDPS and 89 published signatures. In CGGA-693 cohort, MLDPS exhibited the highest AUC values in predicting overall survival for 1-year (Fig. 5E), 3-year (Fig. 5F) and 5-year (Fig. 5G). Moreover, the AUC values of MLDPS ranked the first or top in CGGA-325 and TCGA cohorts (supplementary Fig. 3). In summary, the above results demonstrated that MLDPS possessed a distinctly superior performance and better extrapolation potential than other prognostic signatures.

**Fig. 6:**
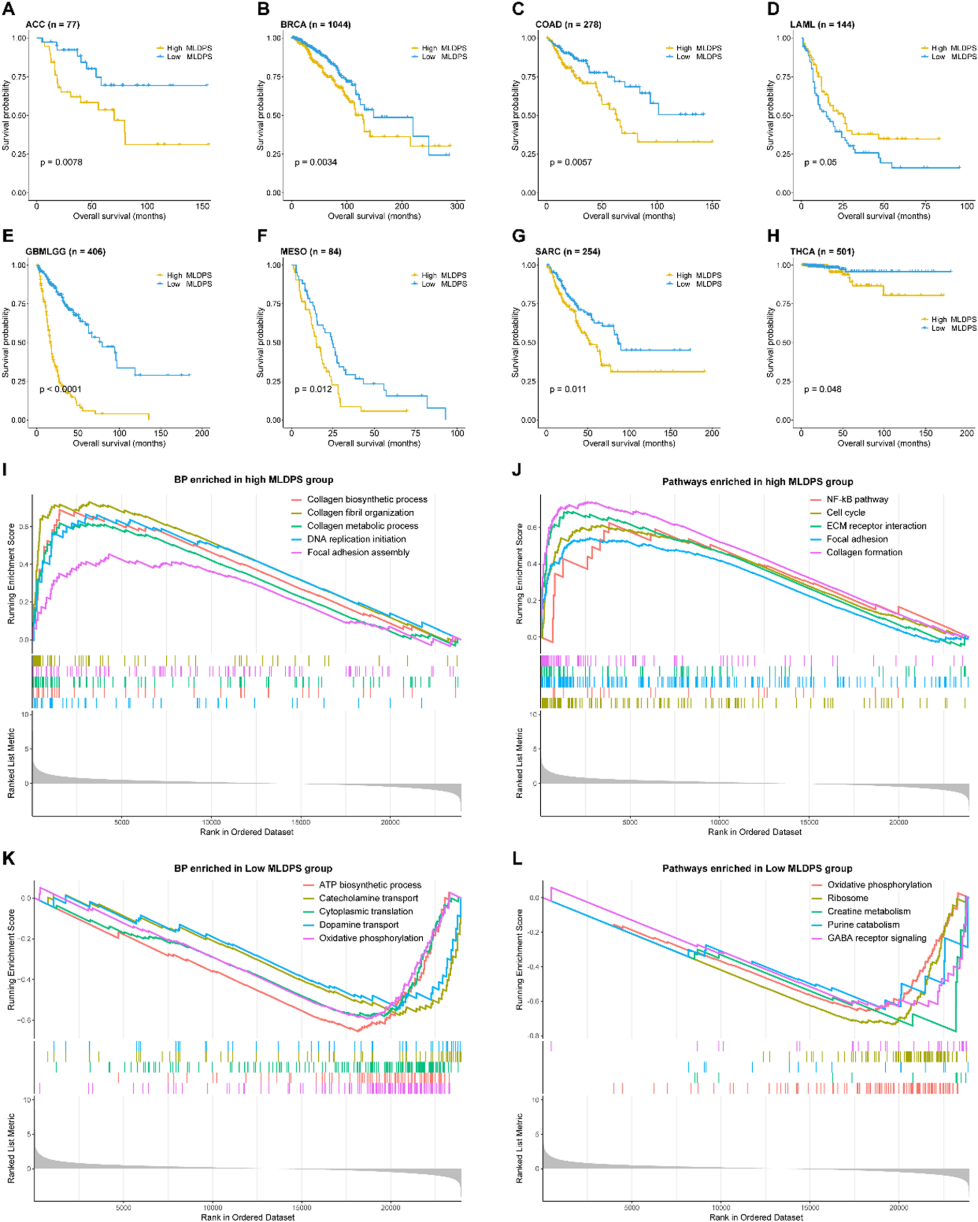
Pan-cancer survival analysis and functional characteristics of the high and low machine learning-derived prognostic signature (MLDPS) groups. (A-H) Kaplan-Meier survival analysis for overall survival (OS) in TCGA-ACC (A), TCGA-BRCA (B), TCGA-COAD (C), TCGA-LAML (D), TCGA-GBMLGG (E, excluded the AYAs glioma), TCGA-MESO (F), TCGA-SARC (G) and TCGA-THCA cohort (H). (I-J) The biological processes (BP) (I) and pathways (J) enriched in high MLDPS group. (K-L) The biological processes (BP) (K) and pathways (L) enriched in low MLDPS group.

Moreover, given the robust predictive performance of MLDPS in AYAs glioma, we additionally evaluated its prognostic value across pan-cancer level. The process included the evaluation of MLDPS in AYAs cancers and the evaluation of MLDPS in cancers with all age groups. After preprocessing, merging and eliminating patients without survival information, OS less than 30 days (these patients may die due to lethal complication such as severe infection and hemorrhage^53,54^, overlapped patients with our AYAs glioma cohort, 9,062 patients from 32 cancer types were included for survival analysis. There were six cancer types with more than 50 patients in AYAs including breast invasive carcinoma (BRCA), cervical squamous cell carcinoma and endocervical adenocarcinoma (CESC), pheochromocytoma and paraganglioma (PCPG), skin cutaneous melanoma (SKCM), testicular germ cell tumors (TGCT) and thyroid carcinoma (THCA). Interestingly, there were no evident association between MLDPS and the prognosis in theses cancers (Supplementary Fig. 4A-F), which might be due to the small sample sizes in these cancer types. Additionally, in cancers with all age groups, the Kaplan-Meier curves indicated that patients in high MLDPS groups exhibited dismal prognosis in adrenocortical carcinoma (ACC, *p* = 0.0078, Fig. 6A), BRCA, (*p* = 0.0034, Fig. 6B), colon adenocarcinoma (COAD, *p* = 0.0057, Fig. 6C), glioma at other age groups (*p* < 0.0001, Fig. 6E), mesothelioma (MESO, *p* = 0.012, Fig. 6F), sarcoma (SARC, *p* = 0.011, Fig. 6G) and THCA (*p* = 0.048, Fig. 6H). While intriguingly, the opposite trend with strong tendency was observed in patients with acute myeloid leukemia (LAML, *p* = 0.05, Fig. 6D), which was probably due to the compositions of ECM in blood cancer differed significantly from those in solid cancers. The above results suggested that MLDPS had potential for generalization to other cancer types.

### The potential biological functions of MLDPS

GSEA analysis was utilized to elucidate the potential signaling pathways and biological processes between different MLDPS groups. As shown in Fig. 6I-J, tumor aggressiveness-related biological pathways were enriched in the high MLDPS group, such as DNA replication initiation, collagen biosynthetic process and formation, NF-kB pathway, cell cycle and focal adhesion. While the low MLDPS group was significantly correlated with metabolism-related functions like creatine metabolism, purine catabolism and oxidative phosphorylation (Fig. 6K-L). Additionally, we also investigated the potential biological functions related to MLDPS. The results indicated that MLDPS was remarkably enriched in cell migration, cell motility, cell adhesion, angiogenesis, extracellular structure organization processes, PI3K-Akt signaling pathway, TGF-beta signaling pathway, ECM-receptor interaction and focal adhesion (supplementary Fig. 4G-H).

### Immune landscape correlated with MLDPS

**Fig. 7:**
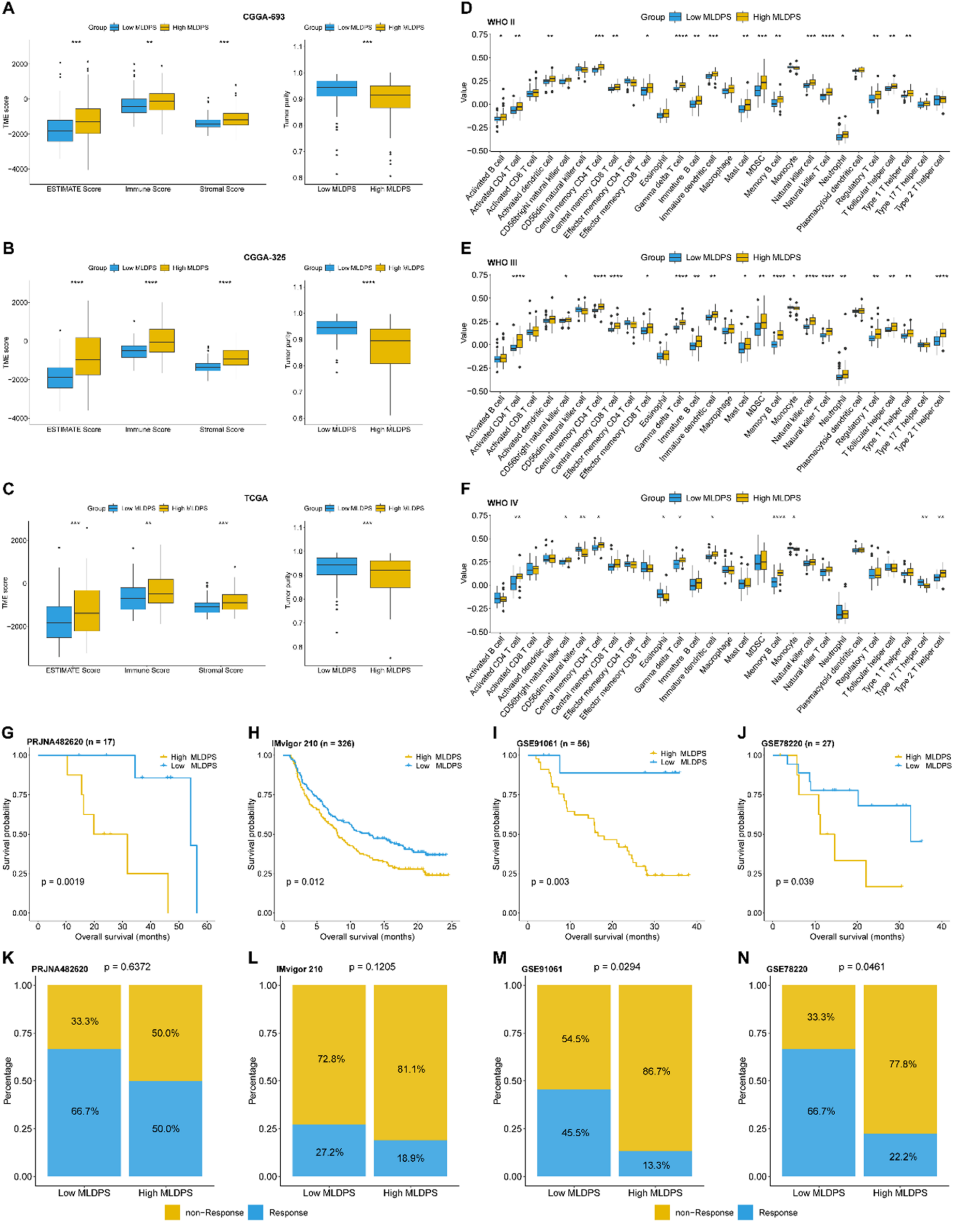
Immune microenvironment analyses. (A-C) The differences in ESTIMATE score, immune score, stromal score and tumor purity between high and low MLDPS groups in CGGA-693 (A), CGGA-325 (B) and TCGA cohorts (C). (D-F) The differences in immune cells between high and low MLDPS groups according to WHO II (D), WHO III (E) and WHO IV (F) in CGGA-693 cohort estimated by ssGSEA method. (G-J) Kaplan-Meier survival analysis for evaluating prognosis in patients received immunotherapy in PRJNA482620 (G), IMvigor 210 (H), GSE91061 (I) and GSE78220 cohort (J). (K-N) The stacked histogram shows the differences in immunotherapy responsiveness between high and low MLDPS groups in PRJNA482620 (K), Imvigor 210 (L), GSE91061 (M) and GSE78220 (N). *p < 0.05, **p < 0.01, ***p < 0.001, ****p < 0.0001.

To elucidate the connection between immune landscape and MLDPS in AYAs glioma, we explored the relationship between MLDPS and different immunity-related indexes. Firstly, we used the ESTIMATE algorithm to calculate immune scores, stromal scores and tumor purity. As shown in Fig. 7A, the high MLDPS group possessed higher ESTIMATE, immune and stromal scores compared to the low MLDPS group in CGGA-693 cohort. The same results were observed in CGGA-325 (*p* < 0.05, Fig. 7B) and TCGA cohorts (*p* < 0.05, Fig. 7C), respectively. These results implied that high MLDPS group owned more infiltration in both stromal and immune cells, which contributes to more complex TME. Additionally, the analyses in tumor purity revealed that high MLDPS groups had remarkably lower tumor purity than low MLDPS groups in CGGA-693 (*p* < 0.05, Fig. 7A), CGGA-325 (*p* < 0.05, Fig. 7B) and TCGA cohorts (*p* < 0.05, Fig. 7C), respectively, which were in harmony with the analyses in above TME scores.

Subsequently, we utilized the ssGSEA algorithms to assess tumor-infiltrating immune cells toward patients with different grades in CGGA-693 cohort. As shown in Fig. 7D, patients in high MLDPS group harbored significantly higher gamma delta T cells, natural killer T cells, T follicular helper cells, neutrophils, activated CD4 T cells and myeloid-derived suppressor cells (MDSCs) than low MLDPS groups in WHO II subgroup. Similar results were observed in patient with WHO III grade (Fig. 7E). Moreover, patients with WHO IV grade in high MLDPS group manifested with obviously elevated memory B cells, immature dendritic cells, activated CD4 T cells, central memory CD4 T cells, gamma delta T cells, which is consistent with the results in WHO II and WHO III subgroups (Fig. 7F). While CD56dim natural killer cells, eosinophils and monocytes were prominently increased in low MLDPS group (Fig. 7F). In addition, the CIBERSORTx analyses revealed that high MLDPS group had obviously fewer resting NK cells than low MLDPS group in WHO II subgroup, while neutrophils and M2 macrophages were evidently higher in high MLDPS group in WHO III subgroup (supplementary Fig. 5). These results depicted a distinctive immune cell infiltration landscape toward high and low MLDPS groups among patients with different grades.

### Predictive value of MLDPS in immunotherapy

Given that the high and low MLDPS groups possessed different tumor immune microenvironment, we speculated that there might be differences in immunotherapy response between patients with high and low MLDPS. As shown in Fig. 7G, the Kaplan-Meier curve demonstrated that in PRJNA482620, a cohort that included glioblastoma patients who received anti-PD-1 therapy, patients in low MLDPS group had better prognosis (*p* = 0.0019). Similarly, advanced urothelial carcinoma patients in low MLDPS group exhibited better prognosis after PD-L1 therapy in the IMvigor 210 cohort (*p* = 0.012, Fig. 7H). As expected, low MLDPS group of patients received anti-PD-1 therapy had better prognosis in two advanced melanoma cohorts including GSE91061 and GSE78220 (*p* < 0.05, Fig. 7I-J). Furthermore, the stacked histogram indicated that patients in low MLDPS group were more likely to respond to immunotherapy in PRJNA482620 cohort (Fig. 7K), IMvigor 210 cohort (Fig. 7L), GSE91061 cohort (Fig. 7M) and GSE78220 cohort (Fig. 7N). Overall, the above results implied that MLDPS had the potential to be a prognostic signature for evaluating prognosis in patients receiving anti-PD-1/PD-L1 therapy including glioblastoma.

## Discussion

The WHO CNS5 has presented a more detailed classification guideline for CNS tumors in 2021, including highlighting the biological and clinical differences between gliomas with adult-type and pediatric-type group^3^. Unlike the above two well-defined groups, AYAs glioma possess adult-type and pediatric-type features, however, the degree of overlap remains ill-defined^4^. AYAs gliomas contribute to the major cause of cancer-related deaths in AYAs cancer and the mortality in these patients is rising^5,6^. Nevertheless, AYAs glioma remain an understudied population owing to the gaps in medical care and lack of studies concentrating on this population. Meanwhile, there has been no study in developing prognostic signature for survival prediction and clinical management in AYAs glioma as far as we know. To bridge this gap, we developed and validated a reliable and robust independent prognostic signature, named MLDPS, for AYAs glioma based on an integrated machine learning workflow together with circuit training and validation procedure and extracellular matrix-related genes. Apart from the consistent and good predictive accuracy in training and validation cohorts, MLDPS also demonstrated superior performance compared with traditional clinical features and 89 published prognostic signatures based on mRNA data. Moreover, MLDPS had the potential to predict prognosis in patients with other cancer types including ACC, BRCA, COAD, LAML, MESO, SARC, THCA and specially, the glioma at other age groups, revealing the prospects of MLDPS in generalization for cancers. Additionally, the TME varied significantly between high and low MLDPS groups while more immune and stromal cells enriched in high MLDPS group. Furthermore, MLDPS also possessed the capability in evaluating prognosis for patients received immunotherapy. Considering the above results, our MLDPS may be a promising tool for survival stratification and individualized clinical management in AYAs glioma and might serve as a reference study for researchers interested in AYAs glioma.

Recently, numerous studies have constructed the prognostic model for glioma with all age groups. For example, Xu et al.^55^ performed univariate Cox and Lasso analysis to establish an autophagy-related prognostic model for glioma. Using the similar method, Tu et al.^56^ and Zhang et al.^57^ developed a RNA-binding protein-related and cuproptosis‑related prognostic signature in glioma, respectively. These signatures might facilitate the prognosis evaluation in patients with glioma at all age groups. However, when comes to the precise prediction in AYAs glioma, the predictive performance of these models is weak and unstable, which may be due to the low generalization power and overfitting within the models caused by the researchers’ preferences and biases^30^. Accordingly, it is extremely imperative to develop prognostic model with robust generalization ability for AYAs glioma. Two years ago, Liu et al. presented a creative and integrated workflow including ten machine learning algorithms and relevant combinations to improve the predictive accuracy in colorectal cancer^32,33^. This workflow has been employed in a lot of studies which have achieved a superior predictive performance than previous cancer prognostic studies such as pancreatic cancer, ovarian cancer and head and neck squamous cell carcinoma^34,58,59^. It is worth mentioning that despite a higher accuracy has attained via this workflow, overfitting might still exist. Therefore, in order to improve the predictive performance and avoid overfitting as much as possible, we innovatively proposed a circuit training and validation procedure combined with the machine learning workflow, which means each cohort can be a training set while others served as validation. In our study, the highest average C-index was detected in the Ridge model when setting TCGA as the training cohort. Nevertheless, overfitting was observed in this model. Hence, the Ridge model with the second highest mean C-index observed in CGGA-693 training procedure, which reached a consistent and dramatic C-index among all cohorts, was selected as the optimal model. This result demonstrates that the circuit training and validation pipeline has an exceptional capability in avoiding overfitting. We believe this pipeline will be very useful for researchers who want to avoid overfitting in their studies. In addition, compared with 89 published prognostic signatures, MLDPS displayed superior predictive performance, which confirmed the robust value of MLDPS. Specifically, although four published models developed by Li et al.^60^ Pan et al.^61^ Liu et al.^51^ and Zhang et al.^52^ owned higher C-index than MLDPS in TCGA cohort, these models underperformed in other cohorts, which may be due to overfitting and poor generalization ability^34^.

Currently, grade, IDH and 1p/19q status have been widely used in evaluating prognosis and guiding treatment for glioma. Strikingly, obvious improved accuracy was observed when making the comparison between these features and MLDPS. Moreover, higher MLDPS was associated with higher grade, IDH-wildtype and 1p/19q non-codeletion, regarded as the more malignant characteristics in glioma^62,63,^which contributes to interpreting the worse prognosis in high MLDPS group. Several studies have reported the classifications for glioblastoma such as the proneural, mesenchymal, neural, and classical subtypes^64,65^ which potentially varies in prognosis, TME contents, genomic and transcriptomic landscape. We tried to explore the correlation between our MLDPS with these transcriptomic-based classifications. However, the patients with the classification information account for only 5% in the TCGA cohort used in our study. Meanwhile, no transcriptomic-based classifications information was found in both CGGA-693 and CGGA-325 cohorts. This hindered us to compare our results with the reported classifications. In addition, we preliminary evaluated the prognostic value of MLDPS among the pan-cancer level and found that MLDPS had the potential ability in estimating the prognosis of patients with multiple cancer types including ACC, BRCA, COAD, LAML, MESO, SARC, THCA and glioma (excluded the AYAs), indicating that MLDPS not only could predict outcome for AYAs glioma but also glioma patients at other age groups. Moreover, we hope the prospects for generalization of MLDPS might be deemed as a reference study for researchers focused on and wanted to undertake in-depth study in other cancers like breast cancer, which reached the 2.3 million cases and 666,000 deaths in 2022 according to the Global cancer statistics 2022^66^.

Since patients in high and low MLDPS groups had evidently different prognosis, it’s necessary to explore the underlying mechanisms in different groups. The GSEA results suggested that the high MLDPS group was notably enriched in tumor aggressiveness-related pathways like NF-kB signaling pathway^67^. Furthermore, several dysregulated pathways were obviously enriched in the prognostic genes of MLDPS such as PI3K-Akt signaling pathway and TGF-beta signaling pathway^68^. These mechanisms partly explained the worse prognosis and advanced grade in high MLDPS group. In addition, we suppose that the extracellular matrix-related prognostic genes in MLDPS might influence the prognosis in AYAs glioma by activating the signaling pathways, such as NF-kB, PI3K-Akt, TNF and TGF-beta signaling pathway. However, the in-depth mechanisms need further exploration in the future.

The TME comprises multiple components such as endothelial cells, immune cells, stromal cells and ECM^69,70^. In glioma, TME could influence tumor progression, clinical outcome and therapeutic response^71,72^. This study demonstrated that the high MLDPS group possessed higher stromal scores, immune scores while lower tumor purity than low MLDPS group, which prompted us to presume that different proportions of stromal and immune cells in TME might affect prognosis in AYAs glioma. Furthermore, we found that lower tumor purity, which was associated with worse prognosis, was observed in high MLDPS group, consistent with the previous studies focusing on the impact of tumor purity on cancer patient’s prognosis^73–75^. Additionally, various types of immune cells, such as activated CD4 T cells, central memory CD4 T cells, gamma delta T cells and myeloid-derived suppressor cells (MDSCs), were observed highly infiltrated in the high MLDPS group. It has been reported that MDSCs are regarded as a central immunosuppressive factor, thus promoting glioma progression^76^. These results contribute to interpret the dismal prognosis in high MLDPS group.

In the last decade, immunotherapy has revolutionized cancer treatment and brought visible survival benefit for patients in different cancers like advanced non-small cell lung cancer and melanoma^77,78^. However, immunotherapy in glioma has not reached satisfied benefit which may be due to the blood-brain barrier, extensive heterogeneity and immunosuppressive TME^79,80^. Despite these challenges, immunotherapy still emerges as a promising avenue for glioma treatment and many immunotherapies clinical trials for glioblastoma has been conducted worldwide^81^. In our study, patients in low MLDPS group were more likely to respond to the immunotherapy and exhibited better prognosis than patients in high MLDPS group. It has been reported that Type 17 T helper cells (Th17) could be recruited to the glioma tumor environment, thereby converting the tumor environment into an immunologically “hot” environment, which fosters and enhances the antitumor immunity and effectiveness to the ongoing immunotherapy in glioma^82,83^. Therefore, we speculated that the high levels of Th17 cells might be responsible for the better prognosis in the low MLDPS group, however, the underlying mechanisms need to be elaborated in future.

Although MLDPS has promising clinical application value for AYAs glioma, several limitations still exist in this study and need to be noted. First of all, the cohorts used for training and validation were obtained from retrospective studies, large-scale multicenter and prospective cohorts are warranted to confirm our findings. Secondly, the concrete mechanisms of MLDPS in AYAs glioma have not been well elucidated, which requires further study from experiments in vivo and in vitro in the near future. Lastly, to verify the relevance of MLDPS in predicting outcome in immunotherapy, future clinical trials in AYAs glioma receiving immunotherapy are necessary.

## Conclusion

In conclusion, based on multiple machine learning algorithm combinations and circuit training and validation procedure, we developed an ECM-related prognostic signature named MLDPS for predicting prognosis in AYAs glioma. MLDPS not only exhibited robust and stable predictive performance but also outperformed 89 published prognostic signatures. Additionally, patients in different MLDPS group harbored distinct infiltration levels of stromal and immune cells and the low MLDPS group might be more sensitive to immunotherapy. Overall, MLDPS holds great potential in evaluating prognosis and providing precisely individualized clinical management for AYAs glioma.

## Supplementary Information

Below is the link to the electronic supplementary material.

Supplementary Material 1

Supplementary Material 2

## Data Availability

The datasets used in this study has been summarized in supplementary Table 1 and can be accessed through the URL as follows. TCGA, GTEx and pan-cancer cohorts: https://xenabrowser.net/datapages/; CGGA cohorts: https://www.cgga.org.cn/; PRJNA482620, GSE91061 and GSE78220 cohorts: http://tiger.canceromics.org/; ECM-genes: https://pubmed.ncbi.nlm.nih.gov/36915600/; IMvigor 210 cohort: http://research-pub.gene.com/IMvigor210CoreBiologies/.

## Abbreviations

ACC: Adrenocortical carcinoma
AYAs: Adolescents and young adults
AUC: Area under the curve
BRCA: Breast invasive carcinoma
CESC: Cervical squamous cell carcinoma and endocervical adenocarcinoma
CGGA: Chinese Glioma Genome Atlas
CNS: Central nervous system
COAD: Colon adenocarcinoma
DEGs: Differentially expressed genes
ECM: Extracellular matrix
Enet: Elastic network
GBM: Gradient boosting machine
GSEA: Gene set enrichment analysis
GTEx: Genotype-Tissue Expression
LAML: Acute myeloid leukemia
Lasso: Least absolute shrinkage and selection operator
LGG: Low grade glioma
MESO: Mesothelioma
MDSCs: Myeloid-derived suppressor cells
MLDPS: Machine learning-derived prognostic signature
OS: Overall survival
PCPG: Pheochromocytoma and paraganglioma
plsRcox: Partial least squares regression for Cox
ROC: Receiver-operator characteristic
RSF: Random survival forest
SARC: Sarcoma
SKCM: Skin cutaneous melanoma
SuperPC: Supervised principal components
TCGA: The Cancer Genome Atlas
TCGA-GBMLGG: The Cancer Genome Atlas-lower grade glioma and glioblastoma
TGCT: Testicular germ cell tumors
THCA: Thyroid carcinoma
Th17: Type 17 T helper cells
TME: Tumor microenvironment
WHO: CNS5 Fifth edition of World Health Organization tumor classification for CNS
