# Supplementary material for: Machine learning derived development and validation of extracellular matrix related signature for predicting prognosis in adolescents and young adults glioma
Source: Sci Rep. 2025 Aug 7;15:28926. doi: 10.1038/s41598-025-13547-6 (PMC12331974; doi:10.1038/s41598-025-13547-6)
Supplement: Supplementary file 1 — Supplementary Material 1 [file 41598_2025_13547_MOESM1_ESM.docx]

**
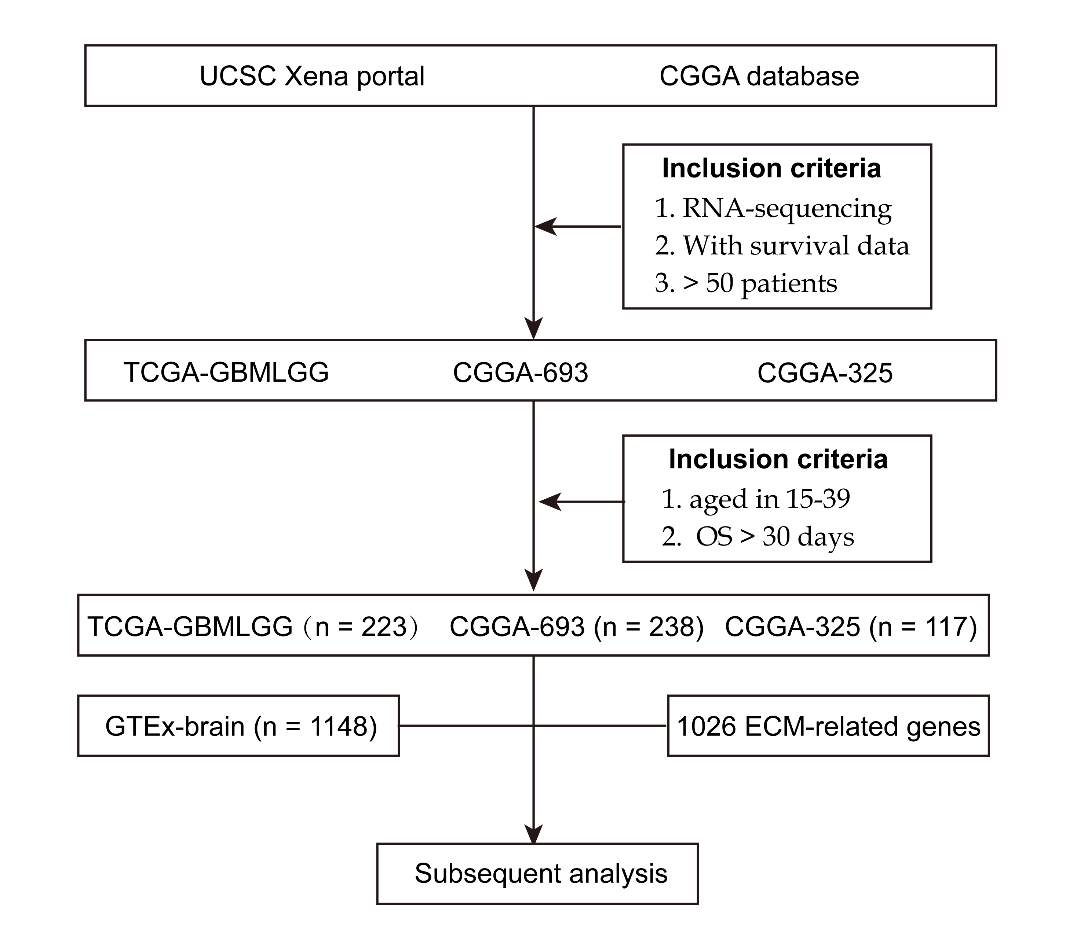
**

**Supplementary Figure 1.** The flow chart for the data acquisition and preprocessing section.


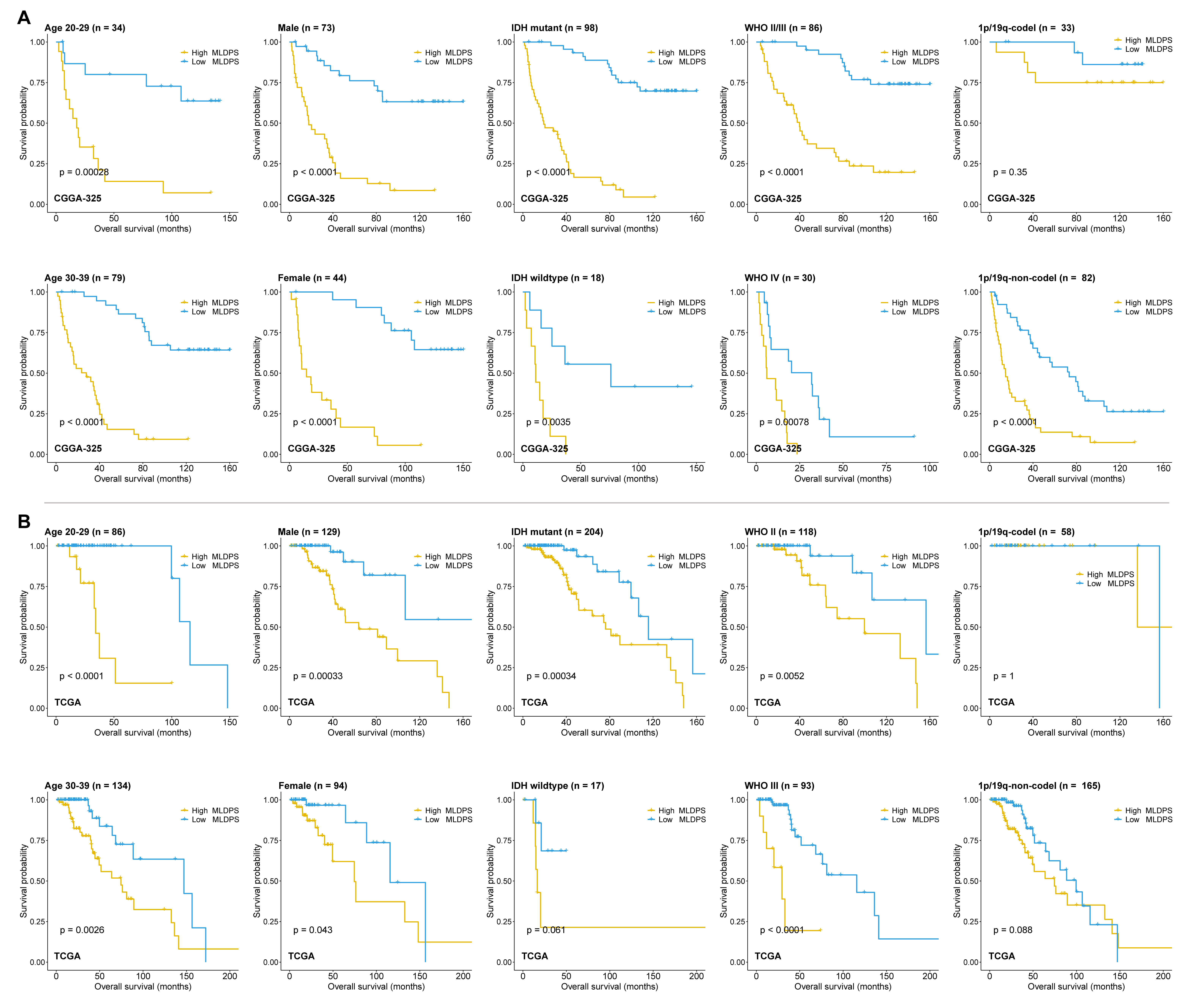


**Supplementary Figure 2.** Subgroup survival analysis. (A-B) Kaplan-Meier survival analysis for overall survival between high and low MLDPS groups in different age, gender, IDH status, grade and 1p/19q status subgroups in CGGA-325 cohort (A) and in TCGA cohort (B), respectively.

**
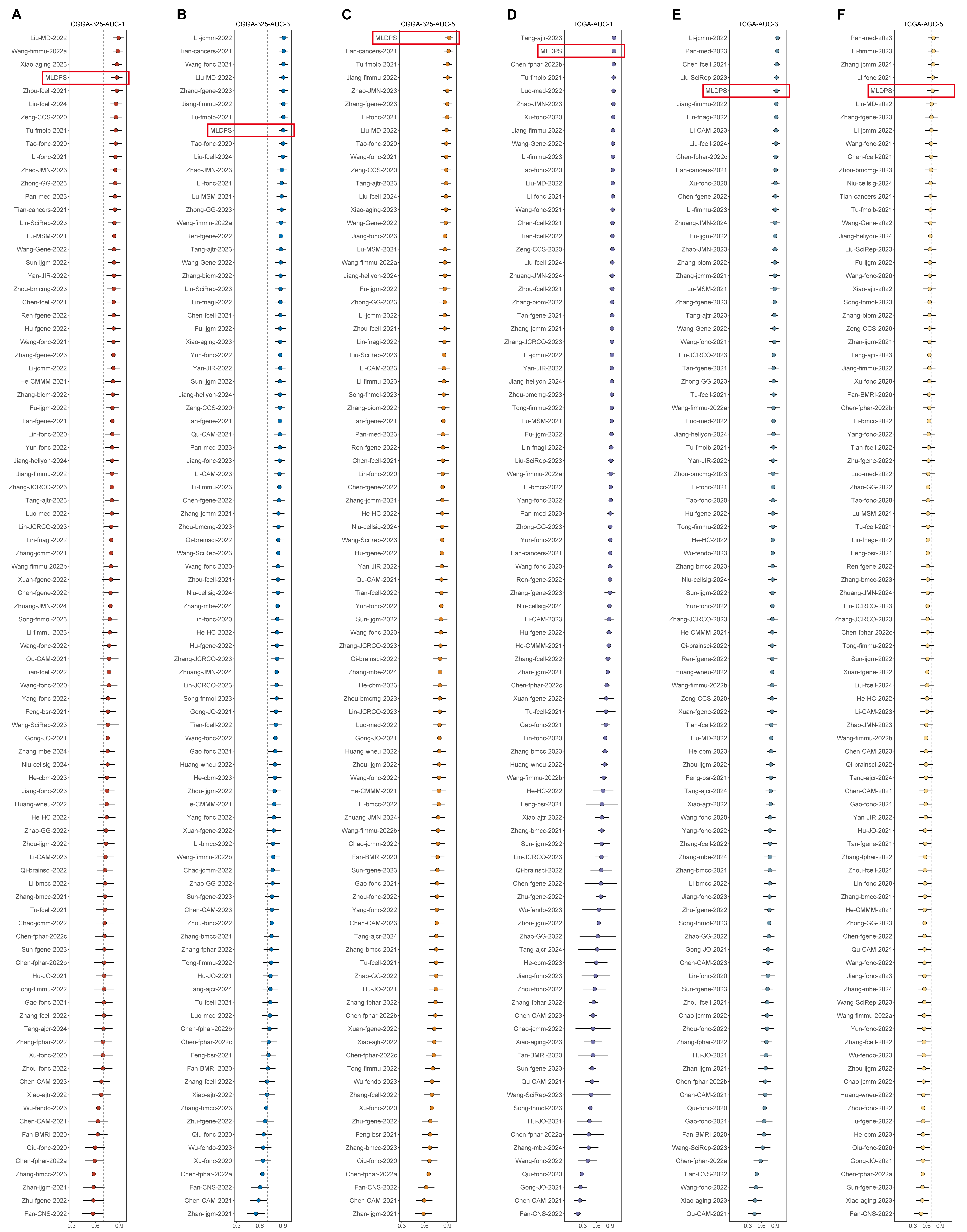
**

**Supplementary Figure 3.** Comparisons between MLDPS and 89 published prognostic signatures. (A-C) AUCs values of MLDPS and 89 published signatures in predicting overall survival at 1-year (A), 3-year (B) and 5-year (C) in CGGA-325 cohort. (D-F) AUCs values of MLDPS and 89 published signatures in predicting overall survival at 1-year (D), 3-year (E) and 5-year (F) in TCGA cohort.


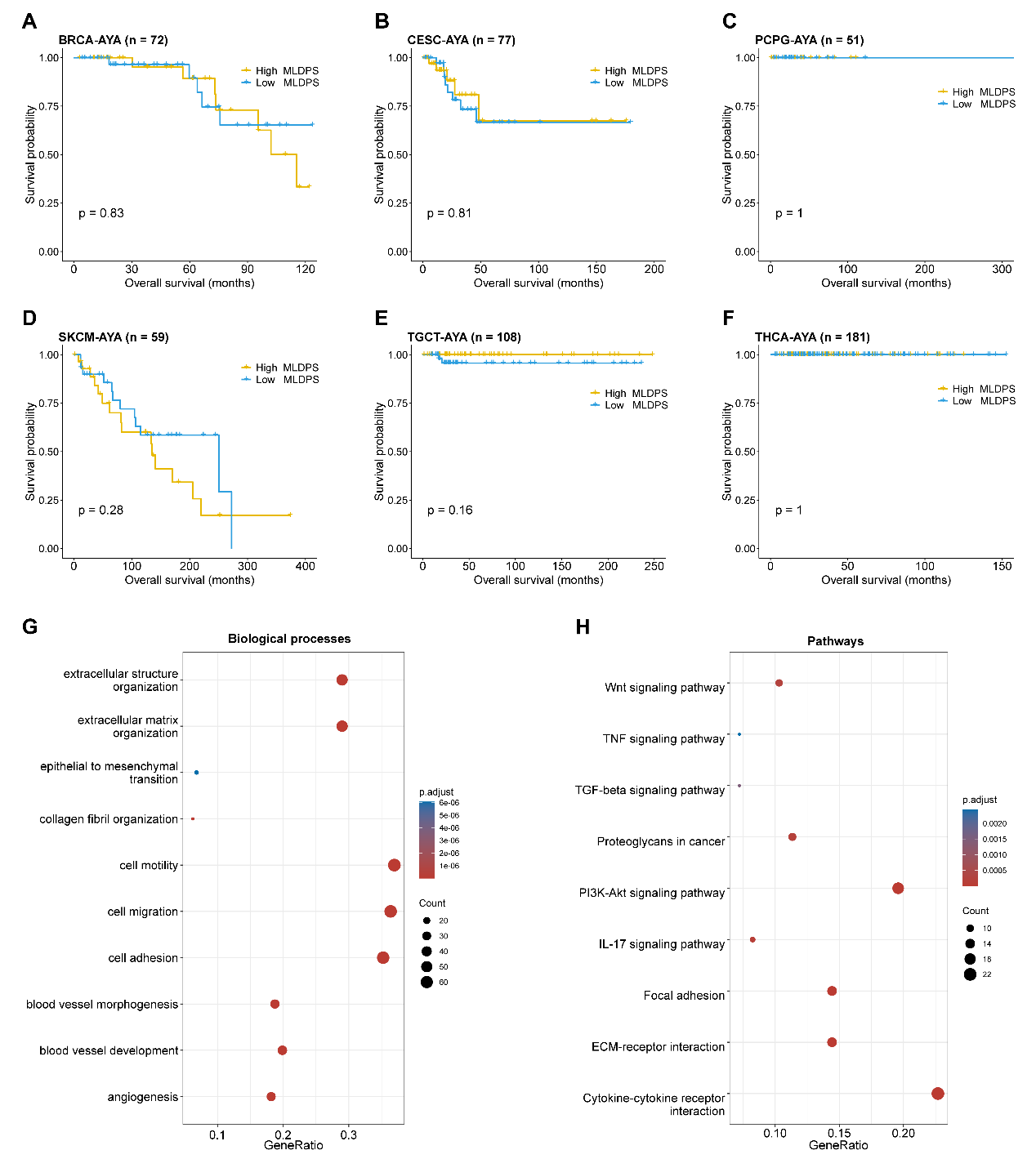


**Supplementary Figure 4.** Pan-cancer survival analysis in adolescents and young adults and functional enrichment analyses of the prognostic genes in MLDPS. (A-F) Kaplan-Meier survival analysis for overall survival (OS) in TCGA-BRCA-AYA cohort (A), TCGA-CESC-AYA cohort (B), TCGA-PCPG-AYA cohort (C), TCGA-SKCM-AYA (D), TCGA-TGCT-AYA cohort (E) and TCGA-THCA-AYA cohort (F). The biological processes (G) and the pathways (H) enriched in the prognostic genes in MLDPS.


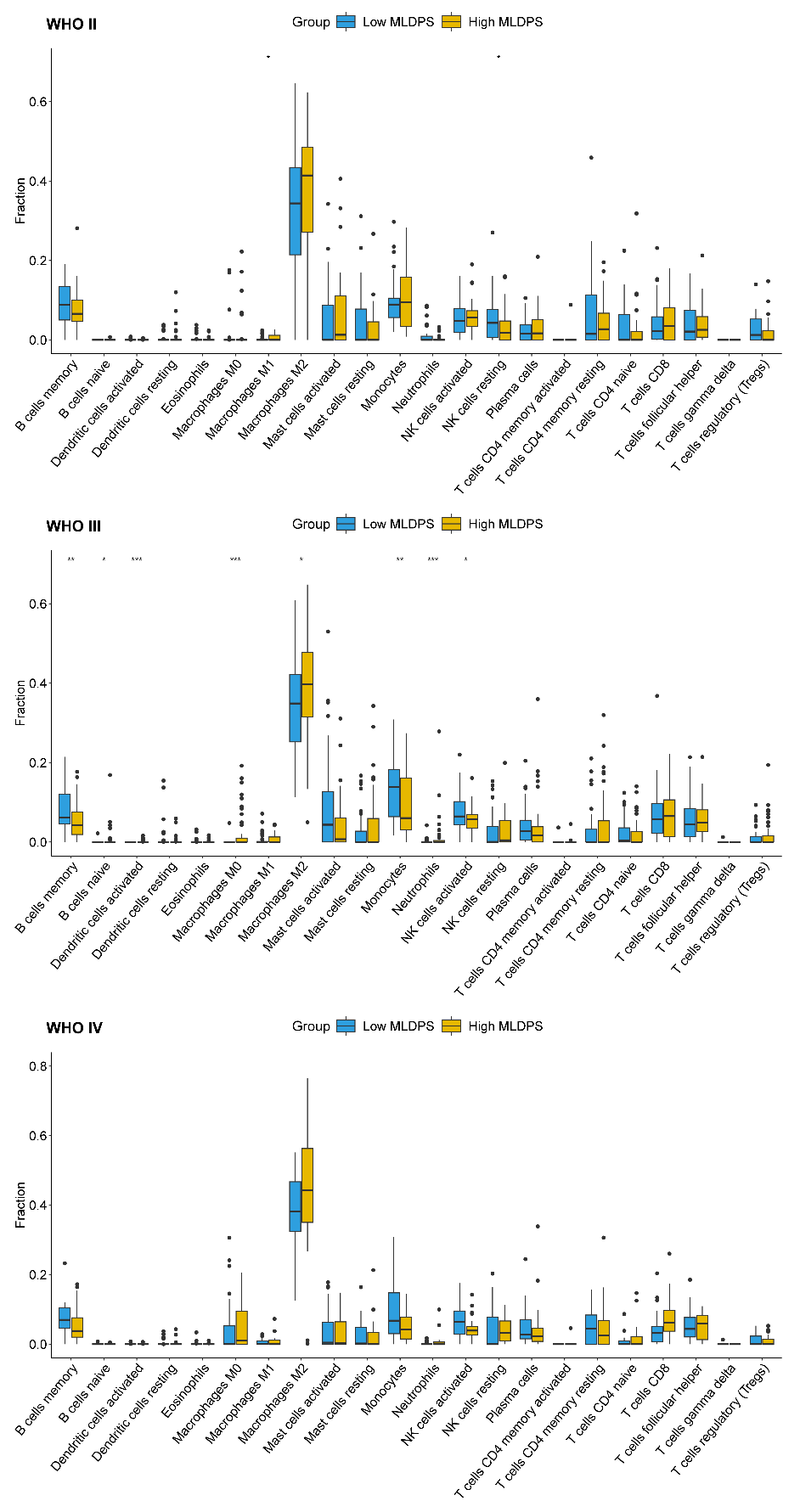


**Supplementary Figure 5.** The differences in immune cells between high and low MLDPS groups according to WHO II, WHO III and WHO IV in CGGA-693 cohort estimated by CIBERSORTx web tool.
